# Effects of organic carbon, inorganic phosphorus, and phosphorus-solubilizing bacteria on maize growth, nutrient uptake, and rhizosphere phosphorus availability

**DOI:** 10.3389/fpls.2025.1644448

**Published:** 2025-08-06

**Authors:** Guangwei Zhou, Mingshuang Wang, Hongxia Zhu, Jing Wang, Shaomin Zhang

**Affiliations:** ^1^ Institute of Agricultural Resources and Environment, Xinjiang Academy of Agricultural Sciences, Urumqi, Xinjiang, China; ^2^ Key Laboratory of Crop Ecophysiology and Farming System in Desert Oasis Region, Ministry of Agriculture and Rural Affairs, Urumqi, China; ^3^ College of Resources and Environment, Xinjiang Agricultural University, Urumqi, China; ^4^ Agricultural Technology Extension Station of Xinjiang Uygur Autonomous Region, Urumqi, Xinjiang, China

**Keywords:** carbon, P-solubilizing bacteria, nutrient uptake, P availability, microbial biomass

## Abstract

**Introduction:**

Phosphate-solubilizing bacteria (PSB), phosphorus(P), and carbon(C )influence the activity of microbes, P availability in soil, and the growth of crops.

**Methods:**

In this study, pot experiments were conducted to evaluate the effects of C, P and PSB on maize growth, nutrient uptake, and P availability in the rhizosphere soil. Based on a 2×3×2 complete factorial design, the pot experiment was performed at two P levels (0 and 50 mg kg^-1^ potassium dihydrogen phosphate), three C levels (0, 60, and 120 mg kg^−1^ glucose) and two PSB levels (0 and 60 mL pot^-1^).

**Results:**

The results showed that PSB addition caused an average increase of 3.03% in the biomass of maize shoots compared to control group with no PSB. C addition resulted in a significant decrease in the biomass of maize shoots, N concentration, and the uptake of nitrogen and P by maize plants. In the absence of exogenous P, PSB addition led to a decrease in N concentration, P concentration, N uptake, and P uptake in maize plants. On the other hand, at exogenous P concentration of 50 mg kg^−1^, PSB addition enhanced N concentration, N uptake, and P uptake in maize plants. The addition of C and PSB led to average decreases of 13.36% and 8.05% in the Olsen P content, respectively, while water-soluble P decreased by 25.52% and 28.42%, respectively. In contrast, microbial biomass C content showed average increases of 78.15% and 60.39%, respectively, while microbial biomass P content increased by 67.52% and 16.19%, respectively.

**Discussion:**

The results showed that C and PSB addition increased the immobilization of microbial C, P and the reduced forms of labile P susceptible to leaching. On the other hand, PSB and exogenous P promoted plant growth by increasing nutrient uptake. The findings of this study will be helpful in promoting the rational use of P fertilizers, reducing P leaching and increasing crop yield.

## 1 Introduction

Maize (Zea mays L.) is one of the main crops in the world, which is grown in more than 130 countries as food and livestock feed (Zhang et al., 2023; Djalovic et al., 2024). Global production of maize is close to 1.1 billion tons, accounting for nearly 30% of the total global grain production (Ahmad et al., 2024). Maize is also the most significant cereal crop in China, with a national production of 277.203 billion kg in 2022, accounting for 40% of the national total grain production (Zhu et al., 2024).

P is an important macronutrient required for key metabolic processes in plants, such as cell division, energy production, biosynthesis of macromolecules, membrane integrity, signal transduction and photosynthesis (Rawat et al., 2021). In surface soils, P concentration is generally 50 to 3000 mg kg^-1^ of soil. However, only 0.1% of total P is available for uptake by plants due to precipitation, immobilization, adsorption, and interconversion to organic forms (Zou et al., 1992; Zhu et al., 2018). As a result, the annual demand and use of P fertilizers have increased over the years to maintain a continuous supply of P to plants (Zou et al., 2022). Long-term heavy application of fertilizers leads to accumulation of P in the soil, which may adversely impact the agricultural systems (George et al., 2016). Therefore, for stable and high-yield agricultural production, it is important to improve the availability of soil P and reduce the application of P fertilizer. Furthermore, there is an urgent need to conserve the P resources and reduce environmental pollution.

Microorganisms play a pivotal role in acquisition of nutrients by plants and are involved in various biological processes (Timofeeva et al., 2022). P-solubilizing bacteria (PSB) are beneficial microorganisms that can dissolve insoluble inorganic and organic P (Li et al., 2021). PSB group accounts for 1%-50% of the total P-solubilizing microorganisms and around 40% of the culturable soil bacteria (Chen et al., 2006; Timofeeva et al., 2022). Various PSB strains, such as *Acinetobacter, Agrobacterium, Bacillus, Burkholderia, Pseudomonas, Pantoea*, and *Sinorhizobium* have been reported (Babalola and Glick, 2012; Raj et al., 2014; Istina et al., 2015; Chen and Liu, 2019; Zhang et al., 2019). Application of PSB to improve the efficiency of P fertilizer and promote crop growth has become an effective way to utilize the P resources (De-Bashan et al., 2022). Microbes assimilate soluble P and prevent its adsorption or fixation (Khan and Joergensen, 2009). PSB inoculation can alter the P-acquisition mechanism of plants and enhance the P uptake, thereby increasing the P content in plant organs and concentration of bio-accessible P in soil, which lead to higher crop yield (Rafi et al., 2019).

Soil microbes play an important role in P cycle (Dai et al., 2020). They secrete protons, carboxylates and phosphatases to mobilize organic and inorganic P (Amadou et al., 2021). Furthermore, microbes can also immobilize soil P into microbial biomass P (Zhang et al., 2022). The tendency of microbes to immobilize or mobilize P depends on their activity and inherent C:P stoichiometry in the soil (Stutter et al., 2015). Microbial biomass P is a dynamic form of soil Po that affects P availability for plants via rapidly responding to the changes in soil environment (Khan and Joergensen, 2009). Many studies have shown that under both laboratory and field conditions, the addition of c sources (such as organic matter and glucose) can increase the microbial biomass P content in soil (Lloyd et al., 2016). C addition may promote the transformation of available inorganic P in soil to microbial biomass P (Zhang et al., 2018). Studies have shown that the application of 3% biochar, along with 1% compost and 1% animal manure, can improve the soil carbon pool, microbial biomass, soil health and soil fertility, thereby enhancing the maize yield (Khan et al., 2023a). The application of phosphate fertilizers with acidified organic amendment and phosphate-solubilizing bacteria (PSB) significantly enhanced the bioavailability of P in soil (Abbas et al., 2024).

However, most studies have investigated the effects of the sole application of C source and PSB on maize growth and P availability. Only a few studies have explored the combined application of C and PSB. The objective of this study was to assess the joint effects of c and PSB on maize growth, nutrient uptake, and soil P availability.

## 2 Materials and methods

### 2.1 Site description

The soil was collected from the Cotton Experimental Station (44^°^17′57″N, 86^°^22′6″E) in the Xinjiang Academy of Agricultural Sciences, Manasi County, Xinjiang, China. The basic properties of soil were as follows: soil texture, loam; pH, 8.10; electrical conductivity (EC) of soil-water suspension (1:5), 0.14 dS m^-1^; organic matter, 6.78 g kg^-1^; ammonium nitrogen concentration, 2.56 mg kg^-1^; and nitrate nitrogen content, 32.34 mg kg^-1^; Olsen P concentration, 22.78 mg kg^-1^; available potassium content, 352.42 mg kg^-1^. After sieving (2-mm sieve), 1.7 kg of soil was filled into each plastic pot, with 13.5 cm height and 15.5 cm diameter.

### 2.2 Experimental design

**Figure 1:**
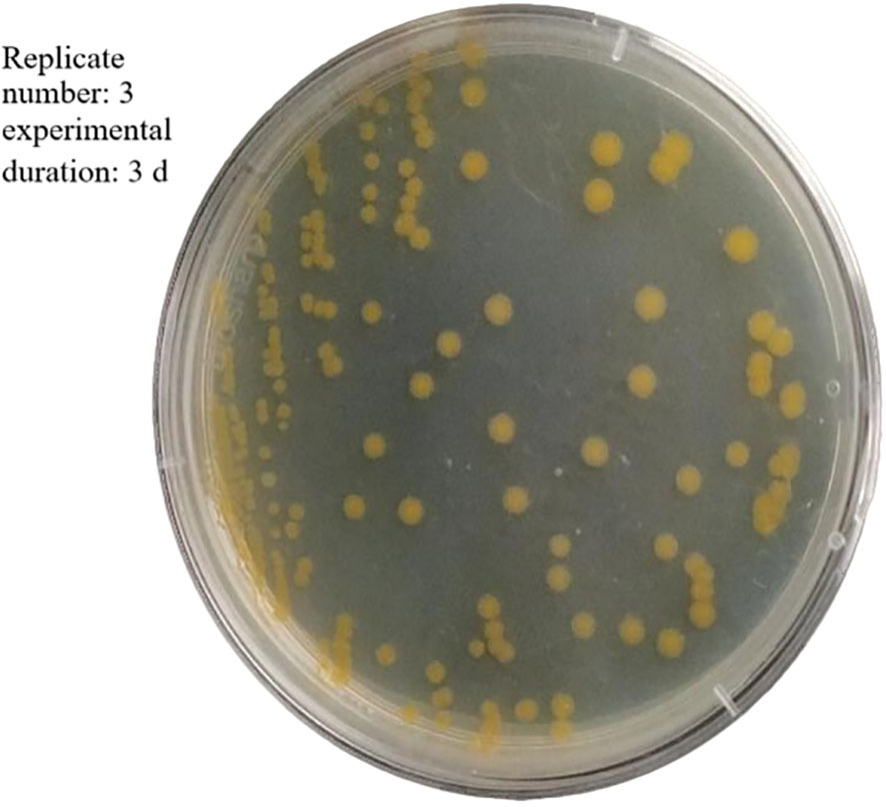
P solubilizing bacteria in LB medium.

The pot experiment was based on a 2×3×2 complete factorial design. A completely randomized block experiment was adopted. Each treatment was repeated three times, using a total of 36 pots. The experiment included two P levels (0 and 50 mg kg^-1^ potassium dihydrogen phosphate), three C levels (0, 60, and 120 mg kg^-1^ glucose), and two PSB levels (0 and 60 mL pot^-1^) (**Figure 1**). The PSB strain was isolated from the rhizosphere soil of *Salinia salinensis* in Manas County, Xinjiang, China. Based on the results of 16S rDNA sequencing and comparison through the NCBI database, the strain was identified as *Pantoea septica* and named *Pantoea septica* PSB. The effective colony number of PSB strain was 1.2×10^9^ CFU mL^-1^. Potassium sulphate was supplied at the rates of 476 and 232 mg pot^-1^, depending on the P levels in soil. Based on the nutrient requirements of maize, the following nutrients were added uniformly before planting (per kilogram soil): 200 mg of nitrogen (urea), 45 mg of calcium (CaCl_2_), 25 mg of magnesium (MgSO_4_), 3 mg of zinc (ZnSO_4_·7H_2_O), 0.5 mg of copper (CuSO_4_), 1 mg of iron (FeSO_4_·7H_2_O), 0.12 mg of boron (H_3_BO_3_) and 0.01 mg of molybdenum (as (NH_4_)_2_MoO_4_). The pots were placed in the light incubation room in random blocks, and the position of each block was rearranged randomly every two days.

The maize (cv. Xinyu69) seeds were planted in the light incubation room on April 12, 2024. P fertilizer was applied to the soil as a one-time basal fertilizer. C and PSB were introduced into the pots four times: once before sowing and then on days 10, 20 and 29 after sowing, respectively. In the treatment group without PSB addition, inactivated PSB was added to eliminate the effect of medium. Nitrogen fertilizer was applied twice before sowing and 14 days after sowing, respectively, while potash fertilizer was introduced into the pots 14 days after sowing. Before sowing, maize seeds were sterilized with 10% H_2_O_2_ for 20 min and rinsed 10 times with deionized water (Zhang et al., 2021). Ten seeds were sown in each pot (height 13cm, diameter 15cm), which were thinned to three seedlings per pot after 7 days. The pots were weighed every 2 days and deionized water was supplied daily to adjust the soil moisture content to 20% (w w^-1^). Differences in the plant weights across different treatment groups were ignored. During the entire experiment, temperature ranged from 25 to 30°C.

Plants samples were harvested 31 days after sowing. Shoot samples were oven-dried at 70°C, weighed, and then milled to measure the concentrations of N and P in shoots (Saleem et al., 2023). The rhizosphere soil collected after harvest was passed through a 2-mm sieve and divided into two parts (Li S, et al., 2024). One part was air-dried to determine the contents of Olsen P and water-soluble P (Chen et al., 2023). The other part was stored at 4°C and later used for analysis of microbial biomass C and P contents (Gao et al., 2021).

### 2.3 Sampling and measurement methods

Plant samples were taken and digested with concentrated sulfuric acid and hydrogen peroxide (Shi, 1986). N concentration in each shoot sample was measured using an AutoKjeldal Unit (Kjeltec 9, Foss, Denmark) (Montemurro et al., 2025). P concentration in shoot was measured by the molybdenum antimony anti-colorimetric method (Qin et al., 2023). Microbial biomass P content in the rhizosphere soil was extracted using the chloroform fumigation-extraction method (Khan et al., 2023a) and quantified by a modified ammonium molybdate-ascorbic acid colorimetric method (Jackson, 1958). Microbial biomass C content in soil was determined by employing the chloroform fumigation extraction method (Khan et al., 2023a). Olsen P content was measured via extraction with 0.5 M NaHCO_3_ at pH 8.5, followed by molybdo-vanadophosphate method (Huo et al., 2022). Water-soluble P content in rhizosphere soil was measured through colorimetry using malachite green (Rahutomo et al., 2019).

### 2.4 Data analyses

The experimental data were analyzed by SPSS statistical software v.22.0 (SPSS Inc., 1996), using a three-factor random block analysis of variance (ANOVA) at a significance level of 0.05, with three independent variables (i.e., C, P, and PSB application rates). A Duncan’s multiple range test was carried out to determine the significant differences between treatment groups at *P* < 0.05.

## 3 Results

### 3.1 Effects of treatments on the shoot biomass in plants

**Figure 2:**
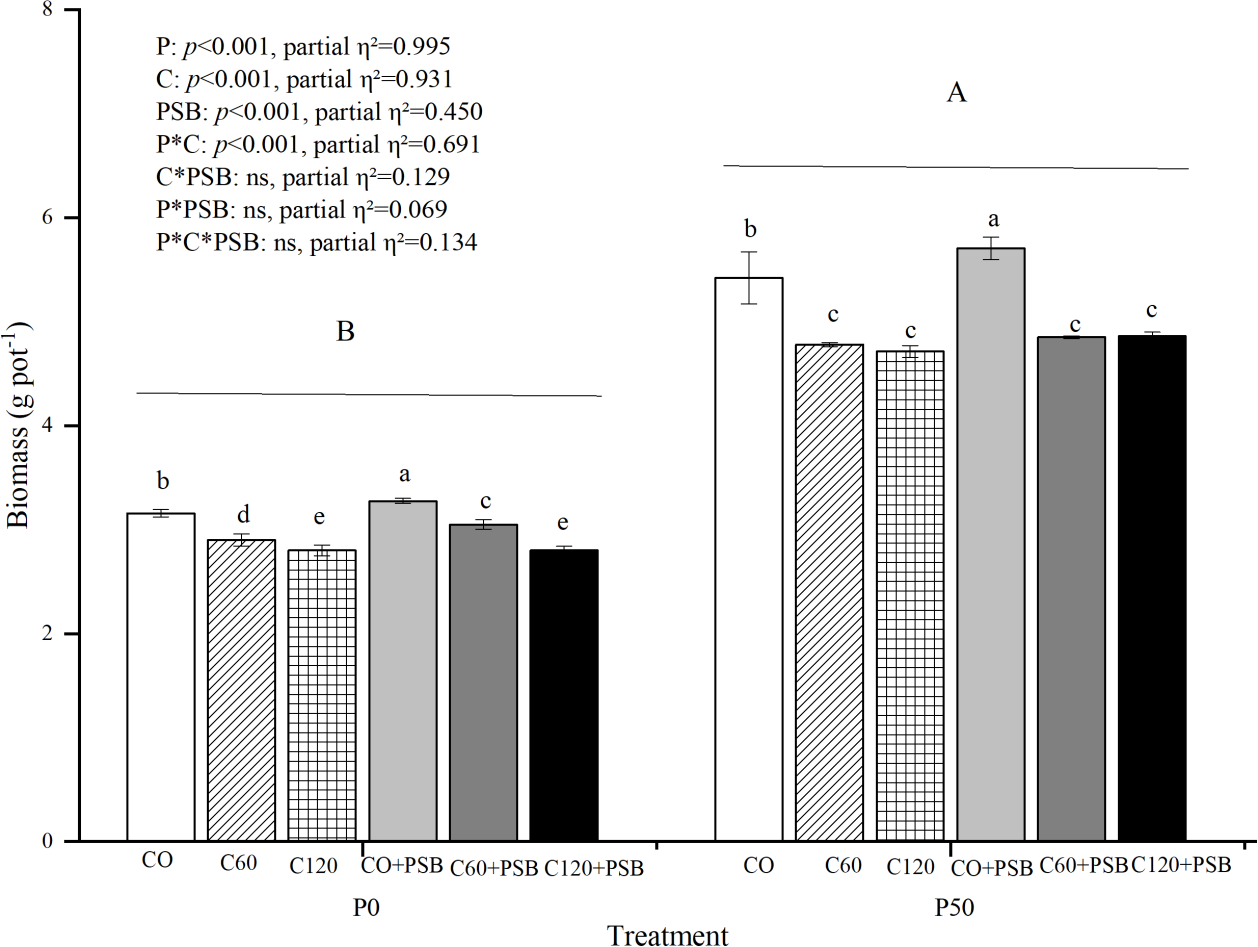
Plant biomass for maize grown in the pot experiment and treated with two P levels (0, 50 mg P kg^−1^), three C levels (0, 60, 120 mg kg^−1^) and two PSB levels (0, 60 mL pot^−1^). Different lower case letters under the same P level indicate a significant difference at different C and PSB addition rates (*p <*0.05). Different capital letter are significantly different at *P* < 0.05 level between P application rates. Bars represent means ± SE.

Plant shoot biomass was significantly affected by the application of C, P, and PSB, as well as the interaction between P and C (**Figure 2**). Plant biomass varied in the range of 2.80-5.71 g pot^-1^. In P0 and P50 groups, shoot biomass decreased with the increase in the C application rate. Compared to P0 group, the P50 group showed a significant increase of 68.86% in the shoot biomass. At both P0 and P50 treatments, the C0+PSB treatment had the greatest values of biomass, 3.28 g pot and 5.71 g pot^-1^, respectively. In the absence of exogenous P supply, C addition caused an average decrease of 11.37% in the plant shoot biomass compared to the group with no C addition. On the other hand, the P50 treatment with added C led to an average decrease of 16.22% in the plant shoot biomass compared to the group with no C. In the P0 treatment, PSB addition resulted in an average increase of 3.03% in plant shoot biomass compared to no PSB addition. On the other hand, the P50 + PSB treatment caused an average increase of 3.14% in plant shoot biomass compared to no PSB.

### 3.2 Effects of treatments on N and P uptake and concentrations of N and P in plants

Application of P, C, PSB, as well as the interactions among P, C and PSB significantly affected the concentrations of N and P in plants, as shown in **Table 1**. The N uptake rate of plants was significantly affected by P and C sole application and interactions, as well as the interaction between P and PSB (**Table 1**). Similarly, P uptake was significantly affected by the application of P, C, and PSB, as well as the interactions of PSB with C and P (**Table 1**). Under P0 conditions, N and P concentration, as well as N and P uptake decreased with the increase in C application rates. Under P50 conditions, plant N concentration and the uptake of N and P decreased with increasing rates of C application, while no significant effect was noticed on P concentration. Under P0 treatment, PSB addition caused an average decrease of 14.71% in plant N concentration compared to no PSB addition. On the other hand, the P50 treatment with added PSB led to an average increase of 3.70% in plant N concentration compared to no PSB. In the P0 treatment group, the addition of PSB addition caused an average decrease of 29.84% in plant P concentration compared to no PSB addition, while plant N uptake decreased by an average of 11.14%. The P50 treatment with PSB addition resulted in an average increase of 6.91% in plant N uptake compared to no PSB. Under P0 treatment, PSB addition caused an average decline of 25.21% in plant P uptake compared to no PSB addition, while the P50 treatment with PSB addition led to an average increase of 2.58% in the plant P uptake compared to no PSB.

**Table 1:** Plant N concentration, P concentration, N uptake, P uptake for maize grown in the pot experiment and treated with two P levels (0, 50 mg P kg^-1^), three C levels (0, 60, 120 mg kg^-1^) and two PSB levels (0, 60 mL pot^-1^).

Treatment			N concentration (g kg^-1^)		P concentration (g kg^-1^)		N uptake (mg pot^-1^)		P uptake (mg pot^-1^)	
P rate	PSB rate	C rate								
P0	-PSB	C0	14.69 ± 0.29a		1.84 ± 0.02a		46.36 ± 0.14a		5.8 ± 0.06a	
		C60	13.95 ± 0.73ab		1.76 ± 0.04a		40.44 ± 1.87c		5.12 ± 0.12b	
		C120	13.72 ± 0.31b		1.52 ± 0.12c		38.42 ± 1.37cd		4.26 ± 0.38c	
	+PSB	C0	13.19 ± 0.60b		1.65 ± 0.03b		43.21 ± 2.27b		5.39 ± 0.10b	
		C60	12.00 ± 0.09c		1.17 ± 0.02d		36.61 ± 0.53d		3.55 ± 0.12d	
		C120	11.73 ± 0.23c		1.13 ± 0.05d		32.85 ± 0.73e		3.17 ± 0.12e	
	Mean		13.21A		1.51B		39.65B		4.55B	
P50	-PSB	C0	14.54 ± 0.09a		2.04 ± 0.02a		78.82 ± 3.88a		11.03 ± 0.42b	
		C60	12.72 ± 0.65b		2.05 ± 0.04a		60.8 ± 2.85c		9.78 ± 0.11c	
		C120	12.52 ± 0.70b		1.98 ± 005a		58.9 ± 2.566c		9.31 ± 0.11c	
	+PSB	C0	14.13 ± 0.20a		2.04 ± 0.05a		80.67 ± 2.65a		11.65 ± 0.37a	
		C60	14.15 ± 0.47a		1.99 ± 0.03a		68.62 ± 2.33b		9.67 ± 0.18c	
		C120	12.97 ± 0.16b		1.97 ± 0.07a		63.01 ± 0.67c		9.58 ± 0.34c	
	Mean		13.51A		2.01A		68.48A		10.17A	
Analysis of variance (*P* value and partial η²)			*P* value	Partial η²	*P* value	Partial η²	*P* value	Partial η²	*P* value	Partial η²
P			<0.001	0.104	<0.001	0.972	<0.001	0.986	<0.001	0.995
C			<0.001	0.652	<0.001	0.847	<0.001	0.922	<0.001	0.945
PSB			<0.001	0.375	<0.001	0.856	ns	0.003	<0.001	0.488
P*C			ns	0.051	<0.001	0.757	<0.001	0.612	ns	0.146
C*PSB			ns	0.105	<0.001	0.563	ns	0.119	<0.001	0.494
P*PSB			<0.001	0.645	<0.001	0.834	<0.001	0.615	<0.001	0.730
P*C*PSB			<0.05	0.302	=0.001	0.421	ns	0.139	ns	0.052

Different lower case letters under the same P level indicate a significant difference at different C and PSB addition rates (*p <*0.05). Different capital letter are significantly different at *P* < 0.05 level between P application rates. "ns" means no significant difference.

### 3.3 Effects of treatments on Olsen P content in soil

**Figure 3:**
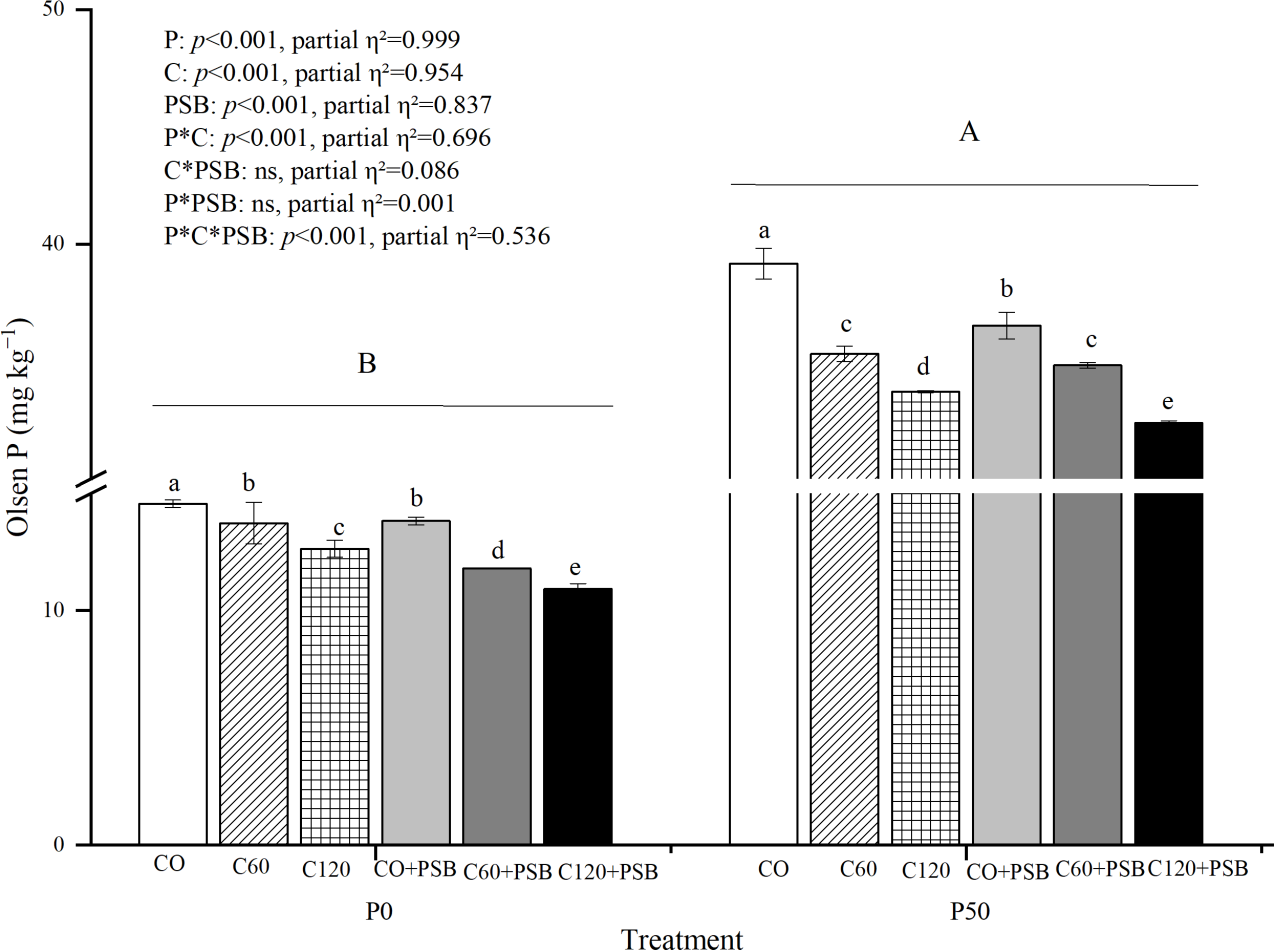
Soil Olsen P for maize grown in the pot experiment and treated with two P levels (0, 50 mg P kg^−1^), three C levels (0, 60, 120 mg kg^−1^) and two PSB levels (0, 60 mL pot^−1^). Different lower case letters under the same P level indicate a significant difference at different C and PSB application rates (*p <*0.05). Different capital letter are significantly different at *P* < 0.05 level between P application rates. Bars represent means ± SE.

Soil Olsen P was significantly affected by the application of P, C, PSB, as well as the interactions among P, C, and PSB (**Figure 3**). In the experimental groups, Olsen P content in soil was in the range of 10.92-39.17 mg kg^-1^. Olsen P tended to decrease with the addition of C and PSB under P0 and P50 conditions. The Olsen P contents in C0 treatment under both P0 and P50 treatment conditions were significantly higher than the other treatments, reaching 14.55 mg kg^-1^ and 39.17 mg kg^-1^, respectively. In the P0 treatment, C addition caused an average decrease of 15.60% in soil Olsen P compared to no c addition, while the P50 treatment with added C led to an average decrease of 11.12% in the Olsen P content in soil compared to no C. Under P0 treatment, PSB addition decreased the Olsen P by an average of 11.96% compared to no PSB addition, while the P50 treatment with added PSB resulted in an average decrease of 4.13% in soil Olsen P content compared to no PSB.

### 3.4 Effects of treatments of the content of water-soluble P in soil

**Figure 4:**
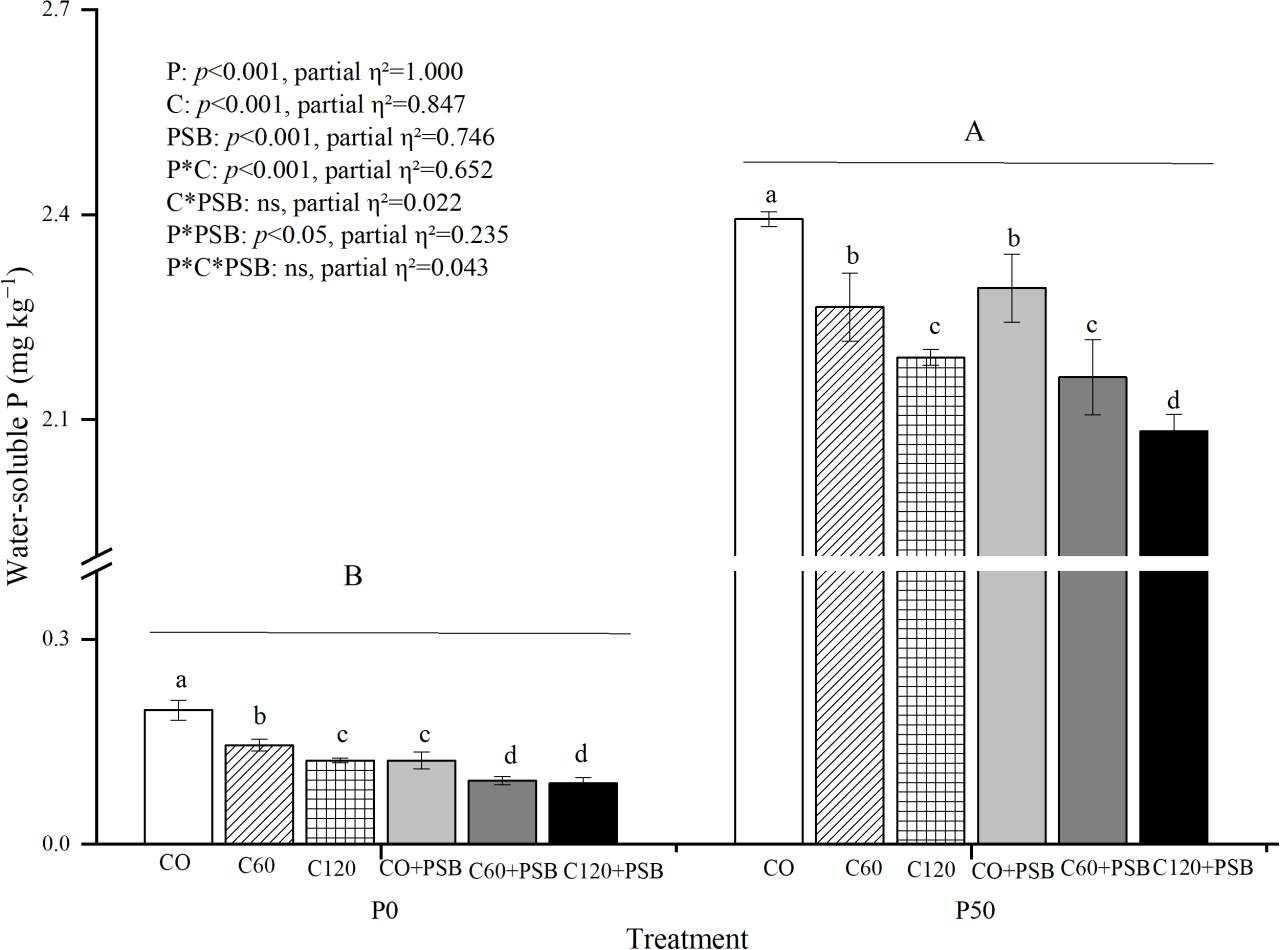
Soil water-soluble P for maize grown in the pot experiment and treated with two P levels (0, 50 mg P kg^−1^), three C levels (0, 60, 120 mg kg^−1^) and two PSB levels (0, 60 mL pot^−1^). Different lower case letters under the same P level indicate a significant difference at different C and PSB application rates (*p <*0.05). Different capital letter are significantly different at *P* < 0.05 level between P application rates. Bars represent means ± SE.

Soil water-soluble P was significantly affected by the application of P, C, and PSB, as well as the interactions of P with C and PSB (**Figure 4**). Across all experimental groups, water-soluble P content in the rhizosphere soil varied in the range of 0.09-2.39 mg kg^-1^. Under both P0 and P50 conditions, the highest water-soluble P content was observed in absence of exogenous C (0.20 mg kg^-1^ and 2.39 mg kg^-1^, respectively) and it tended to decrease with the addition of C and PSB. Under P0 condition, C addition caused an average decrease of 41.71% in soil water-soluble P content compared to no C addition, while PSB addition caused an average decrease of 52.15% compared to no PSB addition. Under P50 condition, C addition led to an average decrease of 9.32% in soil water-soluble P content compared to no C addition. while PSB addition caused an average decrease of 4.69% compared to no PSB.

### 3.5 Effects of treatments on the content of microbial biomass P in soil

**Figure 5:**
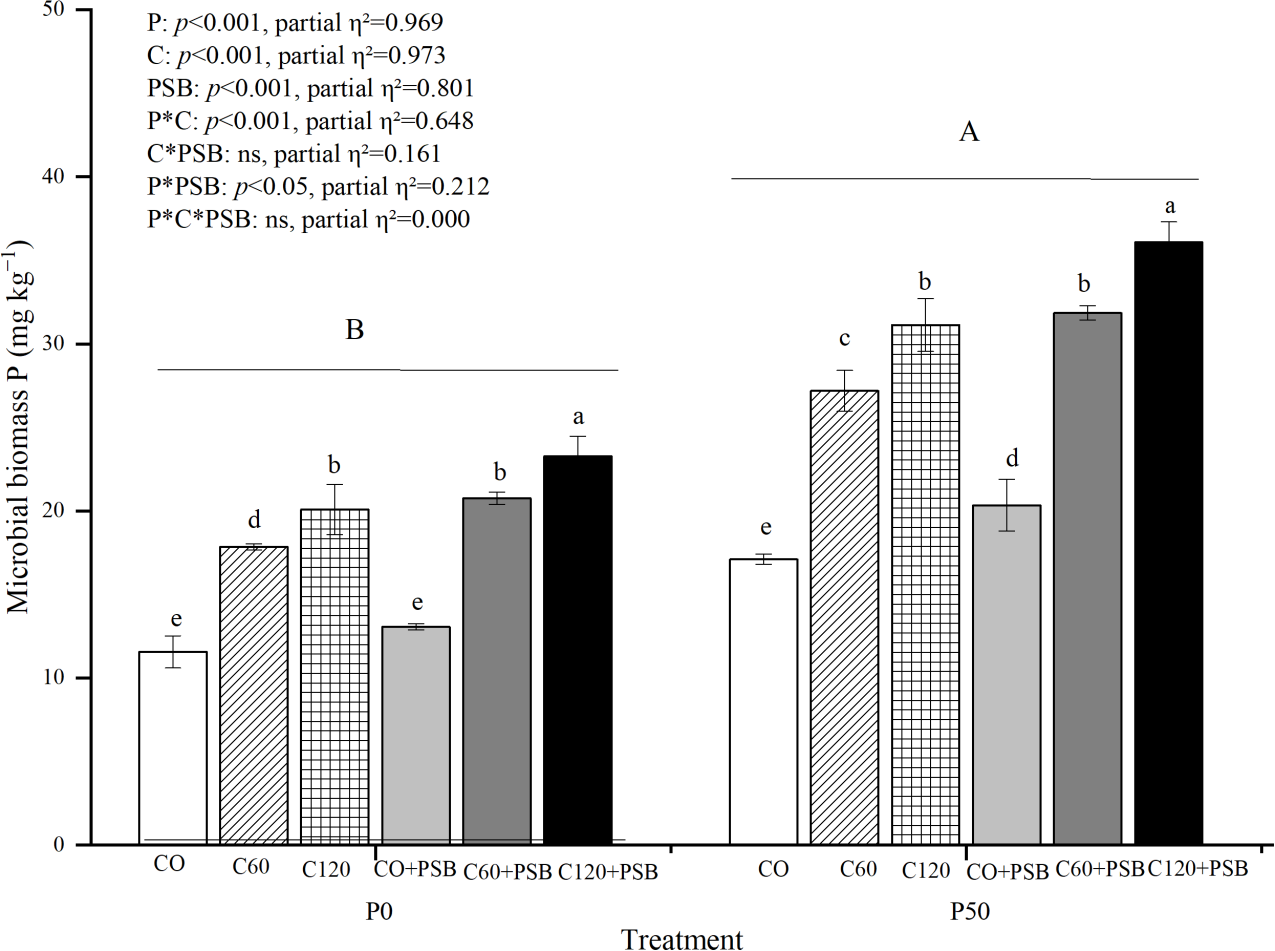
Soil microbial biomass P for maize grown in the pot experiment and treated with two P levels (0, 50 mg P kg^−1^), three C levels (0, 60, 120 mg kg^−1^) and two PSB levels (0, 60 mL pot^−1^). Different lower case letters under the same P level indicate a significant difference at different C and PSB application rates (*p <*0.05). Different capital letter are significantly different at *P* < 0.05 level between P application rates. Bars represent means ± SE.

Microbial biomass P content in soil was significantly affected by the application of P, C, and PSB, as well as the interactions of P with C and PSB (**Figure 5**). Overall, the microbial biomass P content varied between 11.56 to 36.08 mg kg^-1^, and it showed significant increase after application of P, C, and PSB. Under both P0 and P50 conditions, the C120+PSB treatment group showed the highest microbial biomass P contents of 23.29 mg kg^-1^ and 36.08 mg kg^-1^, respectively. Under P0 condition, C addition induced an average increase of 66.46% in soil microbial biomass P compared to no c addition, while PSB addition led to an average increase of 15.32% compared to no PSB addition. Under P50 condition, C addition caused an average increase of 68.57% in soil microbial biomass P compared to no C, while PSB addition enhanced the level of soil microbial biomass P by an average of 17.06% compared to no PSB addition.

### 3.6 Effects of treatments of the content of microbial biomass C in soil

**Figure 6:**
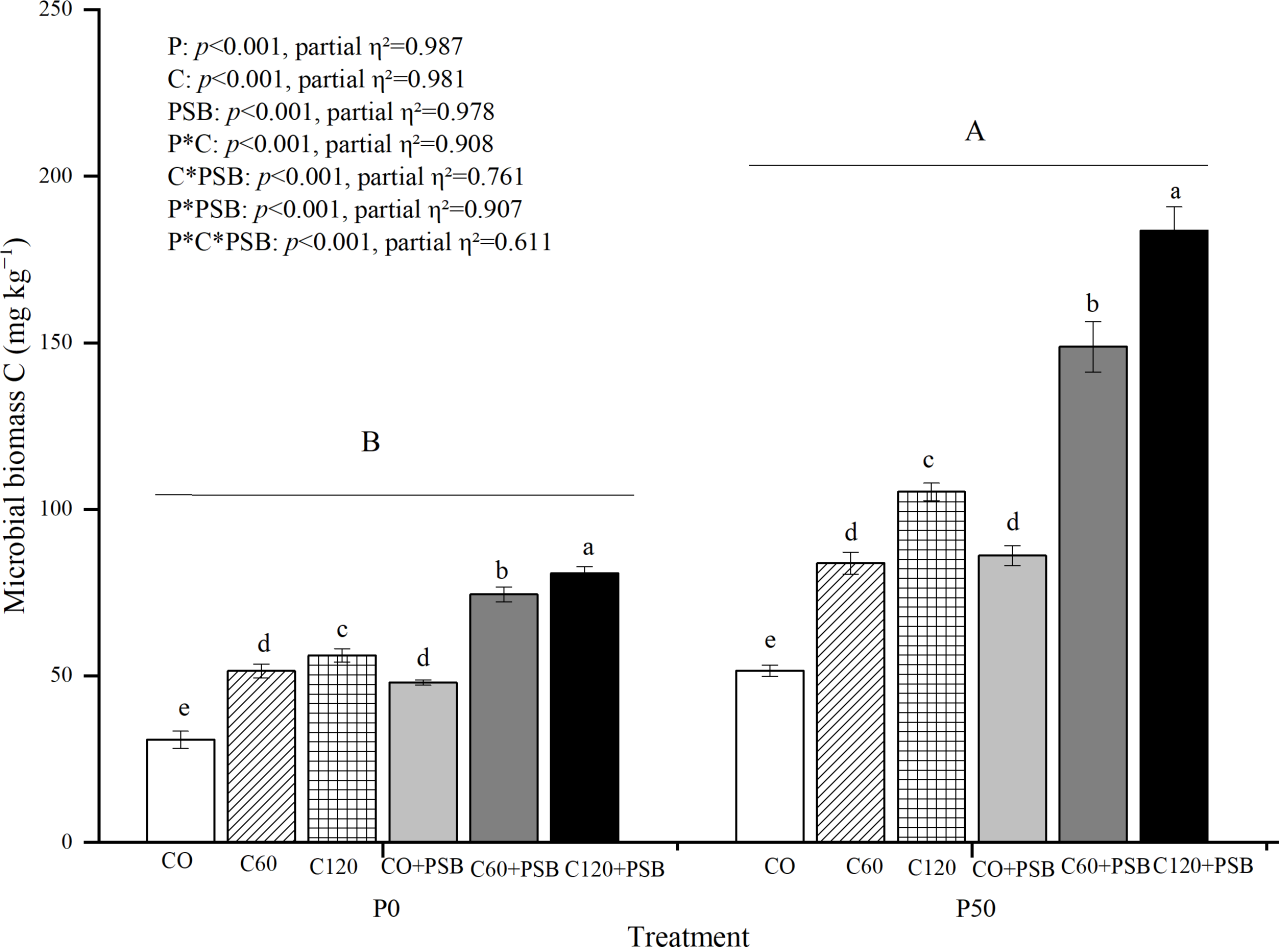
Soil microbial biomass C for maize grown in the pot experiment and treated with two P levels (0, 50 mg P kg^−1^), three C levels (0, 60, 120 mg kg^−1^) and two PSB levels (0, 60 mL pot^−1^). Different lower case letters under the same P level indicate a significant difference at different C and PSB application rates (*p <*0.05). Different capital letter are significantly different at *P* < 0.05 level between P application rates. Bars represent means ± SE.

Soil microbial biomass C content was significantly affected by the application of P, C, and PSB, as well as the interactions among P, C, and PSB (**Figure 6**). Across all groups, soil microbial biomass C content was in the range of 30.83-183.64 mg kg^-1^, showing significant increase with after application of P, C, and PSB. Under P0 treatment, C addition caused an average increase of 66.82% in soil microbial biomass C content compared to no c addition, while PSB addition led to an average increase of 46.84% compared to no PSB addition. Under P50 treatment, C addition resulted in an average increase of 89.47% in soil microbial biomass C content compared to no C, while PSB addition caused an average increase of 73.93% compared to no PSB addition. Under both P0 and P50 conditions, the C120+PSB group showed the highest microbial biomass C contents of 80.83 mg kg^-1^ and 183.64 mg kg^-1^, respectively.

## 4 Discussion

Many studies have investigated the effects of organic fertilizers, biochar, and straw on microbial activity, community composition, and biomass in agricultural soils (Khan et al., 2023b; Bai et al., 2024). These organic amendments increase microbial activity by providing more C (Shu et al., 2022). Only a few researchers have paid attention to PSB strains, which are environmentally safe, inexpensive and highly efficient (Pan and Cai, 2023). In this study, C addition reduced the shoot biomass of maize, which was consistent with the results reported by a previous study (Xu et al., 2020). It is possible that the addition of glucose rapidly stimulated the explosive growth and colonization of soil microorganisms. These microorganisms strongly competed with the plant roots for the limited nutrients in the soil, which affected the uptake of nitrogen and phosphorus by plants, leading to a reduction in crop biomass (Richardson and Simpson, 2011). Although the competition among microorganisms for nutrients was the direct cause, the changes in microbial community structure further amplified this competitive effect. Carbon input promotes the proliferation of r-strategist bacteria (such as those belonging to the Proteobacteria phylum). These bacteria have a high metabolic rate, and they can effectively retain inorganic nitrogen (up to 70% or more of the available nitrogen can be consumed within 48 h). At the same time, they inhibit symbiotic microorganisms (such as arbuscular mycorrhizal fungi and nitrogen-fixing rhizobia), thereby weakening the nutrient acquisition ability of plants. Furthermore, PSB addition increased the shoot biomass in absence of any external C supply, which was consistent with previous results (Ahmed et al., 2025). However, the combined application C and PSB rather inhibited the growth of maize plants. This finding suggests that the combination of C and PSB may affect the microorganisms in the soil, which further affects the crop growth. The present study showed that P uptake by maize decreased significantly with the increasing rate of C application, which was in agreement with the findings of previous research (Xu et al., 2020). Furthermore, PSB addition significantly increased the P uptake in aboveground parts of maize under P50 conditions. This finding was consistent with a previous study, which reported that PSB inoculation significantly improved the growth and P uptake capacity of maize (Alamzeb et al., 2024). However, C and PSB applications had no significant effect on the P content in aboveground biomass of maize at the P50 level. This finding indicates that C and PSB likely stimulate plant growth through other mechanisms besides enhancing P uptake.

Microbial biomass is an important temporary pool of immobilized P that can be mineralized and released into the soil as available P (Wang et al., 2023). P immobilization to microbial biomass by soil microbes and turnover of microbial biomass are considered dynamic transformation processes (Li Y, et al., 2024). This study showed that microbial biomass C and P contents increased significantly with increasing C application rates. This is consistent with previous research, which revealed that glucose addition significantly increased the concentrations of microbial biomass C and P (Huang et al., 2021). On the other hand, PSB addition significantly increased the contents of microbial biomass C and microbial biomass P in this study, which also agrees with the results of previous studies (Liu et al., 2024). The observations suggest that increasing microbial immobilization of labile P into microbial biomass may reduce the content of excessive labile P in the rhizosphere soil. Therefore, it is critical to understand how to manage soil microbial activity to maximize the fixation of labile P.

Olsen P is used to estimate soil P availability to crops. P accumulation is widespread, and soil Olsen P content above the threshold is considered the onset of P leaching in many intensive agricultural systems (Jalali et al., 2025). In this study, C addition decreased the contents of Olsen P and water-soluble P in soil, which is similar to previous research findings (Mehnaz et al., 2019). Soil microbes have become an increasingly important biological source of innovation for global agricultural systems and represent an enormous untapped resource. Integrating phosphorus-solubilizing microorganisms into agricultural systems presents a promising strategy to reduce dependence on chemical fertilizers, enhance soil health, and encourage more sustainable and resilient agricultural practices (García-Berumen et al., 2024). Previous studies have shown that soil microbial communities, including bacteria and fungi, can reduce P leaching from the soil in some systems (Tran et al., 2020; Li et al., 2023). Thus, the addition of C and PSB may reduce the risk of P leaching, thereby protecting the environment and promoting agricultural sustainability. The CFU viability was not monitored after multiple applications in this study, and the exact magnitude of PSB activity in the soil could not be determined, which potentially affected the results. In this study, the C/N ratio was was not measured, which made it difficult to distinguish whether the microorganisms were predominantly involved in mineralization (releasing nitrogen) or fixation (competing for nitrogen). Therefore, the dynamic changes in soil inorganic nitrogen and nutrient release/fixation trend during organic matter decomposition could not be explained (L’Espérance et al., 2024). The absence of microbial activity baseline made it difficult to quantify the intensity of the impacts of environmental or management measures on microbial activity. Furthermore, this also prevented the assessment of the background biological fertility level of the soil. In future research, these limitations will be addressed.

## 5 Conclusions

This study showed that PSB addition increased the shoot biomass of maize, while C addition led to a decrease in shoot biomass. PSB should be applied with phosphate fertilizer to promote the nutrient uptake by maize plants. The addition of C and PSB decreased the contents of Olsen P and water-soluble P, while the contents of microbial biomass C and P increased, which is important to prevent the excessive P leaching in intensive agricultural systems. The results of this study are of great significance for increasing the maize yields and managing the application of phosphate fertilizers. In the future, the impact of adding C and PSB on phosphorus availability needs to be verified through field experiments. In the future, the monitoring of CFU viability needs to be strengthened during the experiment to obtain more reliable results.

## Data Availability

The raw data supporting the conclusions of this article will be made available by the authors, without undue reservation.
